# Sublethal Photodynamic Treatment Does Not Lead to Development of Resistance

**DOI:** 10.3389/fmicb.2018.01699

**Published:** 2018-07-31

**Authors:** Rawan Al-Mutairi, Artak Tovmasyan, Ines Batinic-Haberle, Ludmil Benov

**Affiliations:** ^1^Department of Biochemistry, Faculty of Medicine, Kuwait University, Kuwait City, Kuwait; ^2^Department of Radiation Oncology, Duke University Medical Center, Durham, NC, United States

**Keywords:** photodynamic, antimicrobial, antibiotic resistance, Zn porphyrin, photosensitizer

## Abstract

A promising new alternative approach for eradication of antibiotic-resistant strains is to expose microbes to photosensitizers, which upon illumination generate reactive oxygen species. Among the requirements for a potent, medically applicable photosensitizer, are high efficacy in killing microbes and low toxicity to the host. Since photodynamic treatment is based on production of reactive species which are potentially DNA damaging and mutagenic, it might be expected that under selective pressure, microbes would develop resistance. The aim of this study was to determine if antibacterial photodynamic treatment with a highly photoefficient photosensitizer, Zn(II) *meso*-tetrakis(*N*-*n*-hexylpyridinium-2-yl)porphyrin would lead to development of resistance. To answer that question, bacterial cultures were subjected to multiple cycles of sublethal photodynamic stress and regrowth, and to continuous growth under photodynamic exposure. Antibiotic-resistant *Staphylococcus aureus* and *Escherichia coli* clinical isolates were also tested for susceptibility to photodynamic inactivation and for development of resistance. Results demonstrated that multiple photodynamic exposures and regrowth of surviving cells or continuous growth under sublethal photodynamic conditions, did not lead to development of resistance to photosensitizers or to antibiotics. Antibiotic-resistant *E. coli* and S. *aureus* were as sensitive to photodynamic killing as were their antibiotic-sensitive counterparts and no changes in their sensitivity to antibiotics or to photodynamic inactivation after multiple cycles of photodynamic treatment and regrowth were observed. In conclusion, photosensitizers with high photodynamic antimicrobial efficiency can be used successfully for eradication of antibiotic-resistant bacterial strains without causing development of resistance.

## Introduction

Increasing resistance of bacteria to antibiotics creates a demand for development of alternative methods for eradication of pathogenic microbes. Antimicrobial photodynamic therapy (aPDT), has shown promising results for treatment of localized infections caused by fungi, Gram-positive and Gram-negative bacteria. The therapeutic protocol is based on the use of a non-toxic, light-absorbing compound, the photosensitizer (PS) and visible light. When illuminated, a PS can acquire a relatively long lasting (microseconds) excited triplet (T1) state (for details see, Benov, 2015). A PS in T1 state can participate in two types of reactions: type I and type II. In type I reaction the PS abstracts an electron from a neighboring molecule, producing radicals. In an aerobic environment, the PS anion radical would donate the acquired electron mainly to oxygen, generating superoxide radical (${\text{O}}_{2}^{•-}$), thus initiating series of reactions producing reactive oxygen species (ROS). Among them are cell-damaging species like hydrogen peroxide and hydroxyl radical (HO^•^) (Benov, 2001). In Type II reaction, the excited PS transfers energy directly to molecular oxygen, converting it to singlet oxygen (^1^O_2_). Type I and type II processes compete, and most PSs generate both radicals and ^1^O_2_. Both, hydroxyl radical generated by type I reactions and singlet oxygen produced by type II process are highly reactive (Fridovich, 2013), but for reasons listed elsewhere (Benov, 2015) ^1^O_2_ is considered to be the primary cell damaging factor for most PSs. Therefore, one of the requirements for efficient PS is high singlet oxygen quantum yield (Φ_Δ_) (Cieplik et al., 2014) defined as the number of molecules of singlet oxygen generated per number of photons absorbed by the PS.

Biological systems lack enzymatic protection against ^1^O_2_ but are well-protected against ${\text{O}}_{2}^{•-}$ and H_2_O_2_. In most bacterial species, including *Staphylococcus aureus* and *Escherichia coli*, superoxide radical is removed by superoxide dismutases (SODs) (Benov and Fridovich, 1994; Fridovich, 2013), and H_2_O_2_ is detoxified mainly by catalases and peroxidases (Imlay, 2008). Bacteria respond to increased levels of ${\text{O}}_{2}^{•-}$ and H_2_O_2_ by transcriptional induction of genes coding for protective proteins, coordinated by the *soxRS* (Demple et al., 1999) and *oxyR* regulons, respectively (Lushchak, 2011; Chiang and Schellhorn, 2012). It is known that bacteria pre-exposed to sub-lethal concentrations of redox-cycling compounds generating ${\text{O}}_{2}^{•-}$, or to H_2_O_2_, can tolerate concentrations of these chemicals that are lethal to naïve cells (Demple and Halbrook, 1983). Therefore, a possible mechanism of increased microbial tolerance against aPDT could be induction of oxidative stress response regulons.

Among the requirements for a medically-applicable PS are high singlet oxygen quantum yield, high photoefficiency, and high selectivity. High singlet oxygen quantum yield is a prerequisite for efficient photodynamic killing, but is not sufficient to determine the biological photoefficiency of a PS. Photoefficiency denotes the ability of a PS to inactivate the highest number of unwanted cells at the lowest possible concentration. It depends on the physico-chemical properties of the PS, including lipophilicity, charges and their position, shape, size, symmetry, and flexibility of the molecule (Ezzeddine et al., 2013; Thomas et al., 2015). Selectivity represents the ability of the PS to kill the targeted cells while sparing surrounding healthy cells or host tissues. An ideal PS for aPDT should kill maximal numbers of pathogenic microorganisms to prevent or limit regrowth of survivors, and should be sufficiently selective for the microbes to induce minimal damage to tissues in the area of infection.

Photosensitizers applicable as an alternative to conventional antibiotics should not only be highly efficient and selective, but also should not provoke induction of resistance against the photodynamic treatment or against antibiotics.

Because aPDT has short duration and is based on attack of multiple cellular targets by photo-generated ROS, the predominant view is that development of resistance to aPDT is unlikely (Kashef and Hamblin, 2017). An observation, however, that some *S. aureus* strains are more tolerant to aPDT than others (Grinholc et al., 2007), and the finding that some clinical isolates demonstrated decreased susceptibility of to aPDT after sublethal photodynamic exposure (Cassidy et al., 2010), point to a need for detailed investigations of bacterial adaptive responses to photodynamic treatment. As mentioned earlier, microbes react to aPDT-induced oxidative stress by upregulating defense systems (Kim et al., 2002; Nakonieczna et al., 2010; Dosselli et al., 2012), and this response might be a cause for increased tolerance toward aPDT. Furthermore, photodynamically generated singlet oxygen and other ROS are potentially DNA damaging and mutagenic (Farr et al., 1986; Benov and Fridovich, 1996; Ruiz-Laguna et al., 2000), which may ultimately lead to generation and selection of resistant mutants (Kashef and Hamblin, 2017). The nature and quantity of reactive species, and the range of cellular components that may be damaged, depend on the physico-chemical properties of the PS, and may influence the ability of microorganisms to develop resistance. Therefore, properties of PSs which determine cellular uptake, distribution, and photoefficiency, may also affect development of resistance or enhanced tolerance to aPDT. This implies that results obtained with a particular PS should not be generalized and in addition to photodynamic activity, dark toxicity, and selectivity, new compounds should be individually tested for development of resistance.

We have previously shown that a porphyrin-based tetra-cationic amphiphilic PS, Zn(II) *meso*-tetrakis(*N*-*n*-hexylpyridinium-2-yl)porphyrin (ZnTnHex-2-PyP), is highly efficient in photo-inactivating microbes (Thomas et al., 2015; Awad et al., 2016; Alenezi et al., 2017; Moghnie et al., 2017). The aim of this study was to investigate the possibility for development of microbial resistance after multiple cycles of sublethal photodynamic exposure or continuous growth of bacteria in the presence of sublethal PS concentrations and low light intensity.

## Materials and Methods

### Photosensitizer and Light Source

**FIGURE 1:**
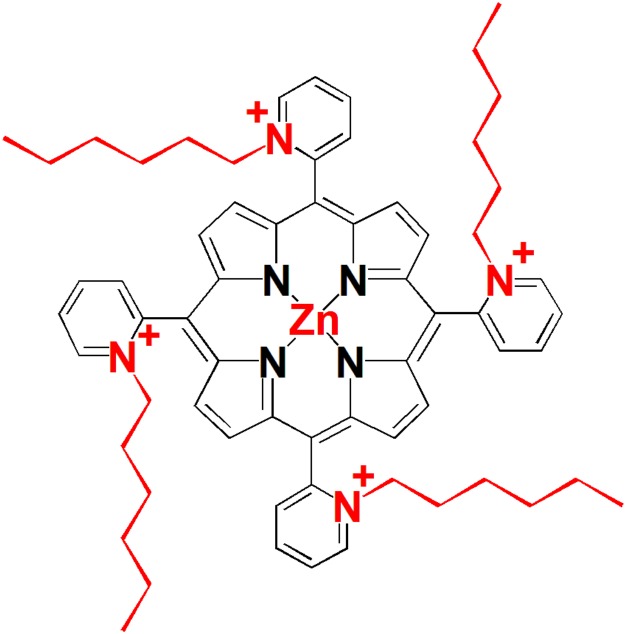
Structure of the photosensitizer used in this study.

Structure of the tetra-cationic Zn(II) *meso*-tetrakis(*N*-hexylpyridinium -2-yl)porphyrin (ZnP, ZnTnHex-2-PyP) is shown in **Figure 1**. Details of the synthesis and characterization of the compound can be found in previous publications (Ezzeddine et al., 2013). ZnTnHex-2-PyP has been chosen because of its high singlet oxygen quantum yield (Φ_Δ_ > 0.85) (Stasheuski et al., 2014), amphiphilic properties, and high antimicrobial photoefficiency (Thomas et al., 2015).

Samples were illuminated in 96-well plates. A broad spectrum light source (overhead projector OHP-3100p, EIKI Industrial, Co. Ltd. with an incandescent 300 W bulb) providing fluence of 37 mW/cm^2^ at 400 nm, was used for illumination of the plates. The emission spectrum of the light source and the absorption spectrum of the PS have been previously published (Thomas et al., 2015). No change in sample temperature was observed during the illumination.

### Strains and Growth Conditions

The following Gram-negative and Gram-positive strains were used in this study: antibiotic- sensitive *E. coli* strain GC4468 (F^-^ Δlac U169 *rpsL*) provided by Dr. D. Touati (Liochev et al., 1999), and a clinical *E. coli* isolate shown to be resistant to carbapenems (provided by Dr. M. John Albert, Faculty of Medicine, Kuwait University, Kuwait); antibiotic-sensitive *S. aureus* strain ATCC25923 (Udo et al., 2000), and antibiotic-resistant clinical isolate CC22-SCC*mec* IV were provided by Dr. E. Udo, Faculty of Medicine, Kuwait University.

Overnight cultures were grown in a shaking water bath at 100 rpm and 37°C in Luria-Bertoni (LB) medium. For preparation of LB plates, 15 g of agar was added to 1 L of liquid LB medium.

For photo-toxicity experiments, the overnight cultures were either diluted 200-fold in LB medium and grown to mid-log phase in a shaking water bath at 200 rpm and 37°C or diluted and immediately illuminated (stationary phase cultures). In all experiments cultures were diluted to the same optical density (OD_600__nm_ = 0.5) to avoid differences in aPDT due to different bacterial densities (Demidova and Hamblin, 2005). In order to prevent photo-generation of toxic metabolites in the medium, illumination was performed in buffered saline (PBS). Experiments were performed in 96-well plates. Portions (100 μl/well) of cell suspensions in PBS were transferred into triplicate wells and ZnTnHex-2-PyP was added to final concentrations indicated in Figure Legends. Unless otherwise stated, after 30 min of incubation in the dark on a shaker at 37°C and 200 rpm, the plates were illuminated for 20 min.

### Viability Assays

Overall effect of aPDT on metabolic activity was determined by the surrogate viability MTT test (Berridge and Tan, 1993; Berridge et al., 2005; Tsukatani et al., 2009). MTT reagent was prepared by dissolving 25 mg of 3-(4,5-dimethylthiazol-2-yl)-2,5-diphenyltetrazolium bromide (MTT) in 5 ml PBS. For solubilization of the formazan crystals, 10% SDS in 10 mM HCl was used. Immediately after illumination, sterile glucose solution (final concentration 0.2%) and 10 μl of MTT reagent were added to all wells and plates were incubated for 30 min in the dark. After 30 min, SDS reagent was added (100 μl/well) and plates were incubated for 1 h at room temperature. The absorbance of each well at 560 and 700 nm (background) was measured using a microplate reader.

Ability of *E. coli* to proliferate was evaluated by plating and counting colonies (CFU assay). After illumination, the samples were diluted and spread evenly on LB agar plates. Plates were incubated for 24/48 h at 37°C in the dark and colonies were counted. Non-treated cultures, dark controls containing PS but not illuminated and illuminated controls not containing PS were assessed the same way.

### Development of Resistance

In order to test the ability of bacteria to develop resistance against aPDT, cultures were treated with sublethal doses of PS and illuminated. Surviving cells were regrown and again treated the same way. This cycle of sublethal photodynamic treatment and regrowth was repeated 10–20 times. After each aPDT treatment, cell responsiveness to aPDT was tested by determining metabolic activity with the MTT assay and cell proliferation by plating and counting colonies. The susceptibility of the treated cultures to aPDT was compared to that of the original, non-aPDT treated strains.

In order to investigate development of resistance in *E. coli* continuously grown under sublethal photodynamic conditions, cultures were grown in 96-well plates for 48 h in LB medium (100 μl/well) in the presence of low concentrations of PS (0.5, 1.0, and 2.0 μM) and low light fluence (0.5 mW/cm^2^) (cumulative light dose for 48 h = 86.4 J/cm^2^). Conditions were selected based on results of preliminary experiments which demonstrated that cultures did not grow if exposed to >2.0 μM of ZnTnHex-2-PyP and light fluence higher than 0.5 mW/cm^2^. The 96-well plates were illuminated on a shaker at 37°C and 200 rpm. After 48 h of incubation, the content of each well (100 μl) was transferred to 5.0 ml of LB medium, cultures were grown to stationary phase, diluted to OD_600_ = 0.5 in PBS and susceptibility to aPDT was tested as already described.

In a separate set of experiments, the surviving cells from the 10-cycle experiments were inoculated in LB medium containing 0, 1.0, or 2.0 μM of ZnTnHex-2-PyP. The cultures were grown for 48 h in 96-well plates under constant illumination at a fluence of 0.5 mW/cm^2^. After that the cultures were tested for susceptibility to aPDT as described above.

In all experiments, untreated controls, controls containing PS but not exposed to light (dark controls), and cultures illuminated in the absence of PS (illuminated controls) were run in parallel. Since no differences between untreated controls and illuminated controls were observed, such data is not shown in part of the figures. Where MTT reduction is presented as a percentage, it is calculated taking MTT reduction of untreated controls as 100%.

### Data Analysis

Each experiment was repeated at least two times with not less than three replicates. Data was analyzed using SigmaPlot 11.0 software, and presented as medians and 25%/75%.

## Results

The purpose of the initial experiments was to determine a sublethal aPDT protocol which would be used subsequently in experiments to testing for development of resistance. Light fluence and time of illumination were selected based on previous study (Thomas et al., 2015). It was found that 20 min of illumination in the presence of 1.0 μM ZnTnHex-2-PyP suppressed metabolism by ∼80% and killed ∼99% of the cells (Supplementary Figure 1). Based on these results, unless otherwise indicated, ZnTnHex-2-PyP was applied at 1.0 μM for performing consecutive cycles of aPDT and regrowth of surviving cells.

**FIGURE 2:**
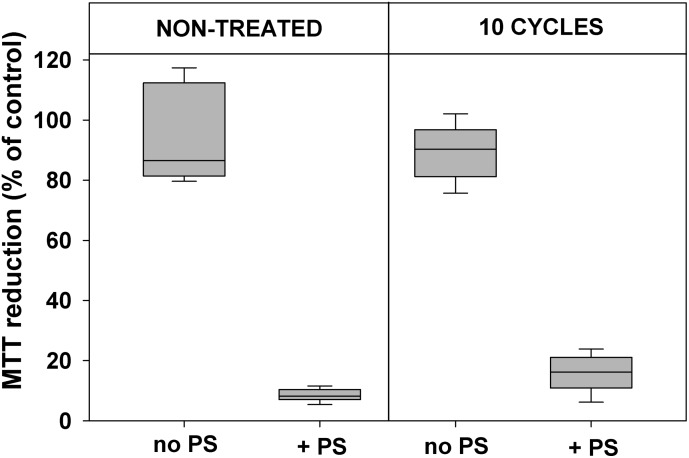
Effect of sublethal photodynamic treatment and regrowth on suppression of cell metabolism by aPDT. After 10 cycles of sublethal photodynamic treatment and regrowth, cells were resuspended in PBS to OD_600_ = 0.5, incubated for 30 min with 1.0 μM ZnTnHex-2-PyP in the dark and illuminated for 20 min at a light fluence of 37 mW/cm^2^. Glucose was added to 0.2% and cell metabolic activity was determined by the MTT test. Cultures grown under the same conditions and subjected to the same treatment except exposure to PS, and original cultures not subjected to any treatment (controls), were tested in parallel. MTT reduction was calculated as a percentage of the formazan product formed by untreated controls. Results of two independent experiments, each sample in triplicate, are presented as medians and 25/75 percentiles.

**FIGURE 3:**
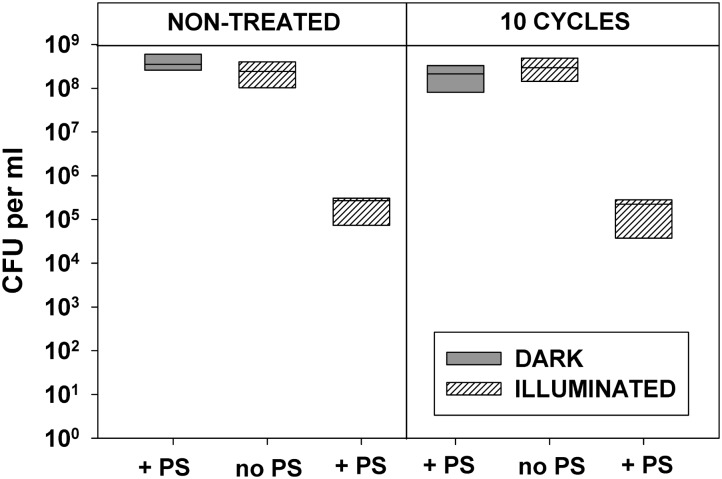
Effect of repeated sublethal photodynamic treatment on *Escherichia coli* ability to replicate. All conditions were as in **Figure 2**. After the last illumination, cells were diluted and evenly spread on LB agar plates for enumeration of colonies (CFU assay). Cultures grown under the same conditions and subjected to the same treatment except exposure to PS, and original cultures not subjected to any treatment (controls), were tested in parallel. Results of two independent experiments, each sample in triplicate, are presented as medians and 25/75 percentiles [colony-forming unit (CFU)] surviving cells.

Data presented in **Figures 2**, **3** demonstrate that 10 cycles of sublethal photodynamic treatment did not lead to development of resistance against aPDT. Irrespective of repeated photodynamic exposure, *E. coli* could not develop protection against aPDT-induced suppression of metabolism (**Figure 2**) or loss of viability (**Figure 3**). Similar results were obtained when the number of cycles of photodynamic treatment and regrowth was increased to 20 (Supplementary Figure 2).

Since contradictory results have been published regarding the sensitivity of mid-log and stationary phase cultures to aPDT (Nitzan et al., 1989; Bhatti et al., 1998; Gad et al., 2004; Banfi et al., 2006; Tavares et al., 2010), both types of cultures were tested. Results demonstrated that logarithmic and stationary phase cultures were equally sensitive to photodynamic inactivation, which is in agreement with earlier reports (Nitzan et al., 1989; Gad et al., 2004; Banfi et al., 2006). No difference between stationary phase and mid-log cultures was observed after 10 cycles of aPDT exposure and regrowth (Supplementary Figure 3). The figure also shows that stationary phase cultures displayed lower level of MTT reduction than the mid-log samples, a finding that can be attributed to lower metabolic activity.

In a separate set of experiments, *E. coli* cultures were subjected to 10 cycles of milder photodynamic stress by decreasing the concentration of ZnTnHex-2-PyP to 0.6 μM. Such treatment suppressed MTT reduction by 50–60%. Repeated photodynamic exposure under such conditions did not cause development of resistance (Supplementary Figure 4).

**FIGURE 4:**
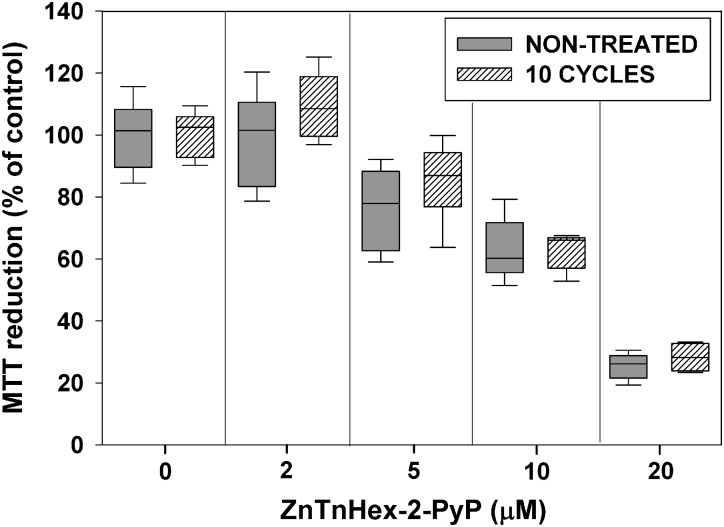
Effect of sublethal photodynamic treatment on dark toxicity of ZnTnHex-2-PyP. After 10 cycles of sublethal photodynamic treatment and regrowth, cells were resuspended in PBS to OD_600_ = 0.5, and incubated in the dark for 50 min with ZnTnHex-2-PyP (2.0–20 μM). Immediately after the incubation, glucose was added to 0.2% and MTT test was performed.

Repeated exposure to sublethal PS concentrations and light doses may upregulate mechanisms preventing uptake of the PS, and as a consequence, may decrease its photoefficiency (Giuliani et al., 2010). To test for such a possibility, after 10 cycles of sublethal aPDT treatment and regrowth, *E. coli* was exposed to ZnTnHex-2-PyP over a concentration range of 2–20 μM, without light exposure. **Figure 4** shows that about 40% suppression of MTT reduction was caused by 10 μM ZnP, and that at 20 μM, the PS suppressed *E. coli* metabolic activity by about 70%. No difference between the naive and the aPDT-treated strains was observed with respect to the dark toxicity of ZnTnHex-2-PyP.

**FIGURE 5:**
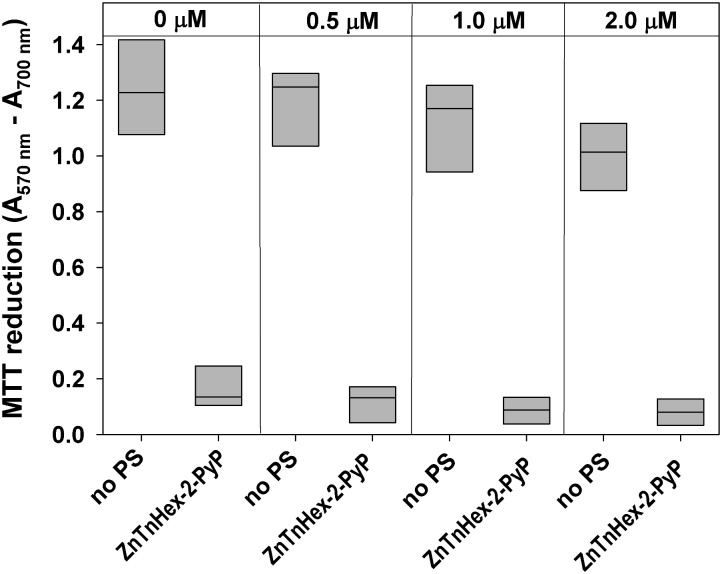
Effect of continuous growth under mild photodynamic stress on susceptibility of *E. coli* to photodynamic inactivation. Overnight cultures were diluted 100 fold in LB medium and allowed to grow for 48 h in the presence of 0.5–2.0 μM ZnTnHex-2-PyP and light intensity of 0.5 mW/cm^2^. Stationary phase cultures obtained that way were diluted in fresh LB medium and regrown. Each sample was then diluted to OD_600_ = 0.5 in PBS, incubated for 30 min with 1.0 μM ZnTnHex-2-PyP and illuminated for 20 min at 37 mW/cm^2^. Reduction of MTT to formazan is presented as a difference in the absorbance at 570 nm (formazan peak) and 700 nm (background). MTT reduction by cultures grown under the same conditions but in the absence of PS is shown for comparison.

Results obtained demonstrated that exposure of *E. coli* to multiple cycles of aPDT treatment and regrowth did not cause development of resistance. However, the experimental design employed, does not expose microbes to conditions similar to those triggering resistance to antibiotics (Kashef and Hamblin, 2017). It has been suggested that a more realistic approach would be sublethal aPDT exposure while allowing bacterial cultures to grow continuously under photodynamic stress (Kashef and Hamblin, 2017). Our investigations demonstrated that *E. coli* cultures inoculated in LB medium containing up to 2.0 μM of ZnTnHex-2-PyP did not grow if exposed to light with fluence of 1.0 mW/cm^2^, but did grow when light intensity was decreased to half of that value. Results show (**Figure 5**) that continuous growth under this level of photodynamic stress did not provoke resistance against aPDT. To confirm that growth under sublethal aPDT conditions does not select resistant mutants, aPDT-treated aliquots were diluted again in LB medium and regrown the same way for 48 h. After three 48 h cycles of sublethal photodynamic exposure, sensitivity of cells to aPDT treatment was tested. Results were not different than those displayed in **Figure 5**.

**FIGURE 6:**
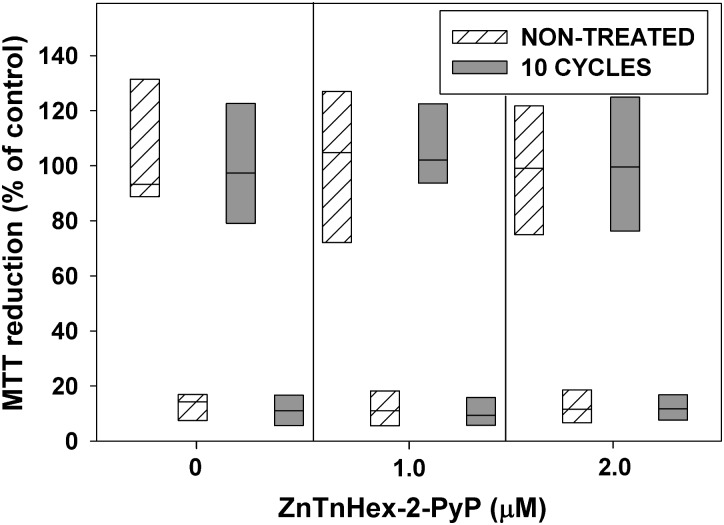
Effect of 10 cycles of sublethal aPDT exposure and regrowth, combined with 48 h growth under mild photodynamic stress, on development of resistance. After 10 cycles of photodynamic treatment and regrowth, cultures were diluted 100 fold in LB medium and allowed to grow for 48 h in the presence of ZnTnHex-2-PyP at final concentrations of 1.0 and 2.0 μM and light fluence of 0.5 mW/cm^2^. Stationary phase cultures obtained that way were diluted to OD_600_ = 0.5 in PBS, incubated for 30 min with 1.0 μM ZnTnHex-2-PyP and illuminated for 20 min at 37 mW/cm^2^. Cultures grown and illuminated in the absence of ZnTnHex-2-PyP were analyzed in parallel. Reduction of MTT to formazan is presented as a percentage of formazan formed by controls not exposed to any treatment. The “X” axis shows ZnTnHex-2-PyP concentrations during the 48 h growth under low light intensity (0.5 mW/cm^2^).

The ability of cells that were exposed to 10 consecutive cycles of photodynamic treatment to develop resistance was also tested by 48 h continuous growth of surviving cells under sublethal photodynamic exposure. **Figure 6** shows that the combination of 10 cycles of sublethal aPDT treatment and growth for 48 h under mild photodynamic stress, did not produce aPDT-resistant mutants.

Resistance to a single antibiotic is frequently accompanied by increased resistance to other antimicrobial agents (Lazar et al., 2013). It could be expected therefore that antibiotic-resistant bacteria might be less sensitive to aPDT than their antibiotic-sensitive counterparts. To test for such a possibility, the photodynamic sensitivity of antibiotic-resistant *E. coli* and *S. aureus* clinical isolates was compared to that of antibiotic-sensitive counterparts. Results demonstrated that photodynamic treatment with ZnTnHex-2-PyP suppressed MTT reduction to the same extent in antibiotic-sensitive and antibiotic-resistant strains (Supplementary Figure 5).

**FIGURE 7:**
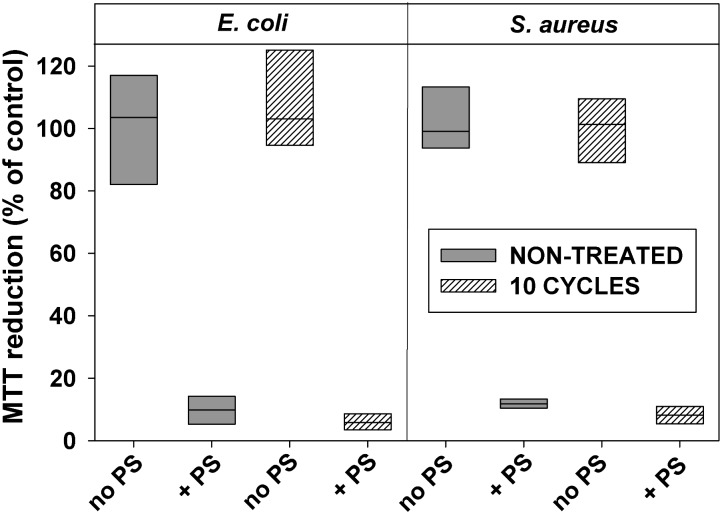
Effect of sublethal photodynamic treatment and regrowth on susceptibility of antibiotic-resistant clinical isolates to inactivation by aPDT. Clinical isolates of *E. coli* and *S. aureus* were subjected to 10 cycles of sublethal photodynamic stress and regrowth. After the 10th cycle, cells were diluted in PBS to OD_600_ = 0.5, incubated 30 min with 1.0 μM of ZnTnHex-2-PyP and illuminated for 20 min at 37 mW/cm^2^. Immediately after the illumination, glucose was added to 0.2% and MTT assay was performed. Cultures grown under the same conditions and subjected to the same treatment except exposure to PS, and original cultures not subjected to any treatment (controls), were tested in parallel. MTT reduction was calculated as a percentage of the formazan product formed by untreated controls. Results of two independent experiments, each sample in triplicate, are presented as medians and 25/75 percentiles.

**FIGURE 8:**
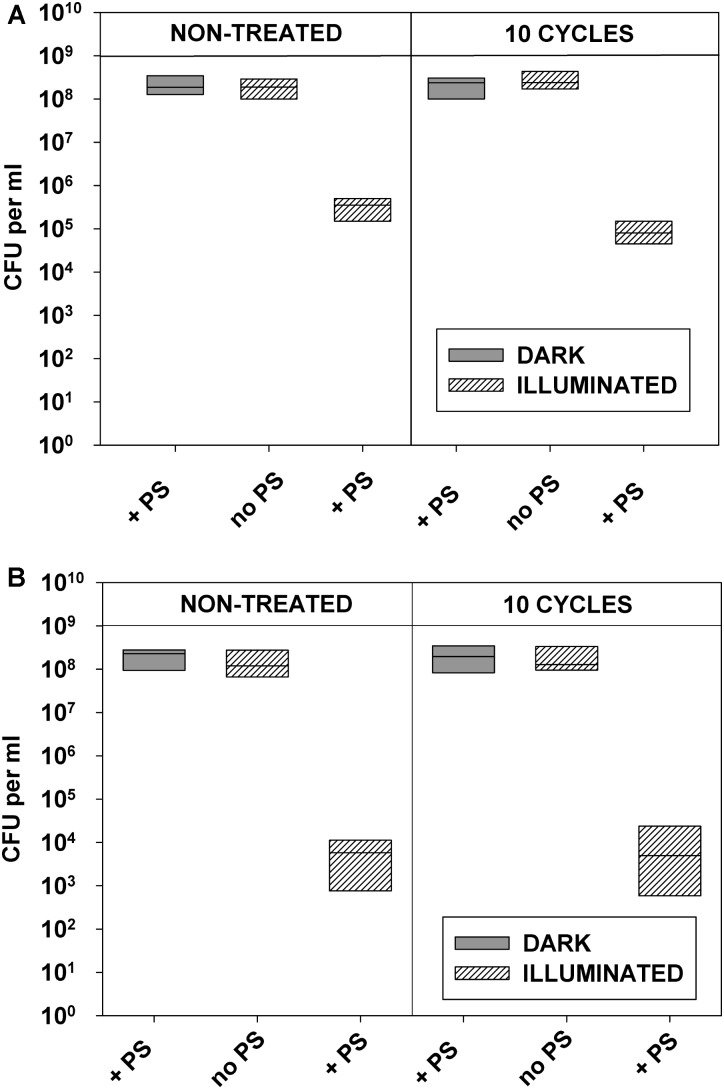
Replicability of antibiotic-resistant clinical isolates exposed to repeated cycles of sublethal photodynamic treatment and regrowth. All conditions were as in **Figure 7**. After 10 cycles of sublethal aPDT exposure, cultures of antibiotic-resistant *E. coli* **(A)** and *S. aureus* **(B)** were diluted to OD_600_ = 0.5 in PBS, incubated for 30 min with 1.0 μM of ZnTnHex-2-PyP, and illuminated for 20 min at a fluence of 37 mW/cm^2^. Immediately after the illumination cultures were suitably diluted, evenly spread on agar plates, and colonies were enumerated.

Similarly to the antibiotic-sensitive strains, clinical *E. coli* and *S. aureus* isolates tested in this study, did not develop resistance after 10 consecutive cycles of sublethal photodynamic treatment and regrowth of surviving cells (**Figures 7**, **8**).

It has been reported that sublethal aPDT can affect antibiotic susceptibility of isolates and the effect is strain-dependent (Kashef et al., 2013). In order to test for such a possibility, after 10 cycles of sublethal photodynamic treatment, susceptibility of bacteria to a range of antibiotics was determined and compared with the susceptibility of untreated controls. Results showed that 10 cycles of sublethal photodynamic treatment did not change the pattern of sensitivity to antibiotics of the clinical *E. coli* and *S. aureus* isolates (Supplementary Table 1).

## Discussion

Excessive use of antibiotics is considered to be the main reason for the spread of antimicrobial resistance (Kashef and Hamblin, 2017). One way to limit the use of antibiotics is, when possible, to apply alternative methods for eradication of microorganisms. Usefulness of aPDT as an alternative to antibiotics requires unequivocal answers to several critical questions:

(a) Can aPDT induce resistance?
(b) Does aPDT affect resistance to antibiotics?
(c) Can aPDT inactivate antibiotic-resistant microorganisms?

Since aPDT is based on production of reactive species whose cytotoxic effects are not limited to a small range of specific targets, it is assumed that development of resistance against photodynamic antimicrobials is unlikely (Maisch, 2015; Kashef and Hamblin, 2017). The possibilities for development of resistance, however, should not be ruled out without detailed investigations of mechanisms of responses and adaptation of microbes to photodynamically induced cell damage.

Results from some studies suggest that exposure to sublethal levels of photodynamic treatment may lead to development of resistance to aPDT and/or to antibiotics (reviewed in Kashef et al., 2013; Kashef and Hamblin, 2017). Differences of aPDT susceptibility among bacterial strains have been reported, but reasons for such differences are not known (Grinholc et al., 2008). No relationship between antibiotic resistance and susceptibility to aPDT has been established (Maisch et al., 2005; Grinholc et al., 2008).

Theoretically, bacteria could be able to develop resistance to aPDT by the same mechanisms they employ to acquire resistance to antibiotics (Blair et al., 2015). It is known that increased mutation frequency leads to development of resistance (Schaaff et al., 2002; Jolivet-Gougeon et al., 2011). Both, hydroxyl radical, produced by type I reactions, and singlet oxygen, generated by the type II mechanism, are potentially DNA damaging (Benov, 2015); this damage can lead to increased mutagenicity (Moody and Hassan, 1982; Epe, 1991) and eventually, generation and selection of resistant mutants.

Another mechanism that can provide enhanced tolerance of bacteria to aPDT is induction of genes coding for protective proteins (Kashef and Hamblin, 2017). Since in aPDT microbes are killed by reactive species generated by photo-excited PS (Hamblin and Hasan, 2004), it has been proposed that enhanced tolerance could result from induction of genes responsible for defense against oxidative stress (Maisch, 2015). Such induction, however, requires a much longer time than the time needed for aPDT treatment (Maisch, 2015). Prolonged PS–microbe interactions can be expected in clinical treatment protocols, where removal of all excess PS is practically impossible (Kashef and Hamblin, 2017). Microbes which survive the photodynamic insult would be exposed to remaining PS and ambient light for a length of time which could be sufficient for selection of resistant mutants or for induction of genes responsible for defense against oxidative stress. We tried to model such conditions by repeatedly exposing *E. coli* to sublethal aPDT doses, re-growing surviving cells, and comparing their sensitivity to aPDT to the sensitivity of cells that were not exposed to any treatment.

Susceptibility of bacteria to aPDT has been assessed by two different methods, the classical CFU assay, and the colorimetric MTT test which measures metabolic activity. The two assays provide information for processes occurring at different periods of the photodynamic treatment. The CFU assay determines the ability of bacteria to replicate. Because it takes substantial time for cells to divide and form visible colonies on agar, the CFU assay provides information only about late consequences of the treatment. Depending on the extent and location of the photodynamic injury, bacterial cells can either recover and survive or die. It has been reported that survival of *E. coli* increases if after illumination cells are transferred to fresh medium (as in plating) (Salmon-Divon et al., 2004). Photodynamic treatment can trigger stress response(s), thus activating repair processes, which depending on the extent of the damage, may reverse the outcome (Salmon-Divon et al., 2004). Reduction of MTT to colored formazan (MTT test) is enzymatically catalyzed and depends on availability of NAD(P)H. Inactivation of metabolic enzymes and respiratory complexes is among the earliest events taking place during photodynamic treatment (Awad et al., 2016). Therefore the MTT test provides an integrated information about the initial cell damage occurring during the illumination.

Results demonstrated that even after 10–20 cycles of aPDT treatment, the surviving cells were as sensitive to aPDT as the non-treated original strains. Such an experimental design, however, has been criticized (Kashef and Hamblin, 2017) because aPDT is a relatively short procedure and microbial cultures were only exposed to photodynamic stress for short periods of time. In contrast to antibiotics that are present during the entire time, in these aPDT-resistance experiments, bacteria that survived were allowed to regrow in the absence of selective pressure. Thus, such experiments fail to model conditions similar to those when microbes acquire resistance to antibiotics where they continuously grow in the presence of low-levels of antimicrobial agents (Kashef and Hamblin, 2017). A better way to test development of resistance to aPDT was proposed in which cultures are allowed to grow continuously under sublethal levels of PS and light exposure (Kashef and Hamblin, 2017).

In this study*, E. coli* cultures exposed for 48 h to 0.5–2.0 μM ZnTnHex-2-PyP and low light fluence (0.5 mW/cm^2^), did grow, and reached stationary phase. When tested, such cultures displayed the same aPDT sensitivity as cultures grown for the same time without photodynamic exposure. Dilution and regrowth of such cultures under the same sublethal conditions for three consecutive 48 h cycles did not produce resistant mutants. Similar results were obtained when after 10 consecutive cycles of aPDT, surviving cells were grown for 48 h under sublethal photodynamic conditions.

Experiments performed with *S. aureus* and Zn (II) phthalocyanine derivative have shown that repeated exposure to the PS, induced resistance to the dark toxicity of the compound (Giuliani et al., 2010). It has been speculated that *S. aureus* developed resistance by altering its membrane structure (Kashef and Hamblin, 2017). In our experiments, repeated or prolonged exposure of *E. coli* and *S. aureus* to ZnTnHex-2-PyP did not affect the tolerance of the microbes for the PS. A reason for this result may be found in the properties of the PS and its cellular targets. Previous investigations have shown that ZnTnHex-2-PyP kills *E. coli* by damaging the cell envelope, without causing detectable DNA modifications (Thomas et al., 2015; Awad et al., 2016). Due to its amphiphilic character and positive charges, ZnTnHex-2-PyP is attracted by anionic membrane components (Epand and Epand, 2009; Alves et al., 2014), and disperses in the lipid bilayer (Kos et al., 2009; Thomas et al., 2015). Upon illumination, the PS generates reactive species directly into the lipid environment, which initiate free radical chain reactions of lipid peroxidation (Alves et al., 2013), leading to life-incompatible membrane damage (Stark, 2005).

Microbes apply various mechanisms to protect themselves against antibiotics, and very often resistance is not limited to a single antimicrobial agent (Kashef and Hamblin, 2017). It can be expected, therefore, that antibiotic-resistant strains are more resistant to aPDT. On the other hand, it has been reported that exposure of antibiotic-resistant clinical isolates to aPDT has altered their antibiotic susceptibility (Kashef et al., 2013). Clinical isolates of antibiotic-resistant *E. coli* and *S. aureus* that were tested in this study did not display resistance to photo-inactivation with ZnTnHex-2-PyP and no increase in resistance to antibiotics as a result of the treatment was observed.

Several reasons can be listed for the inability of bacteria to develop resistance against aPDT. In contrast to classical antibiotics which act with high specificity, ROS generated by photo-excited PSs do not have specific targets. It has been proposed that enhanced tolerance to aPDT may result from better microbial defense against oxidative stress (Maisch, 2015; Kashef and Hamblin, 2017). Previous experiments demonstrated, however, that SOD deficient mutants, where *soxRS* regulon is induced by the higher intracellular steady state concentration of superoxide (Liochev et al., 1999), were as sensitive to aPDT as their SOD-proficient parents (Benov et al., 2002; Thomas et al., 2015). The probability that during the photodynamic treatment bacteria are able to respond by augmenting their defenses against oxidative damage, is very low. Such defense is controlled mainly by the *oxyR* and *soxRS* regulons, and exposure to aPDT is too short to induce these regulons (Liochev et al., 1999). Furthermore, organisms have not developed specific defense systems against singlet oxygen, which is the key cell damaging species produced by photo-excited porphyrins (Benov et al., 2011; Benov, 2015).

## Conclusion

Bacterial populations that survived photodynamic treatment were not capable of developing resistance against aPDT. Prolonged exposure and growth under sublethal concentrations of ZnTnHex-2-PyP and light intensity, and treatment through multiple cycles of exposure to aPDT, did not make bacteria less susceptible to photodynamic inactivation. Sensitivity to antibiotics was also unaffected by the aPDT treatment. Antibiotic-resistant and antibiotic-susceptible bacterial strains were equally sensitive to photo-inactivation with ZnTnHex-2-PyP, which makes aPDT an attractive option for fighting antibiotic resistant microbes.

## Author Contributions

RA-M conducted most of the experiments. AT synthesized the photosensitizer. IB-H designed isomeric Zn porphyrins modifying them to enhance intracellular uptake. LB designed the study, analyzed the data, drafted the manuscript, and prepared the figures. All authors read and approved the final manuscript.

## Conflict of Interest Statement

The authors declare that the research was conducted in the absence of any commercial or financial relationships that could be construed as a potential conflict of interest.
