# Supplementary material for: Sublethal Photodynamic Treatment Does Not Lead to Development of Resistance
Source: Front Microbiol. 2018 Jul 31;9:1699. doi: 10.3389/fmicb.2018.01699 (PMC6079231; doi:10.3389/fmicb.2018.01699)
Supplement: Supplementary file 1 [file Data_Sheet_1.docx]

Supplementary Material

# Sublethal photodynamic treatment does not lead to development of resistance

**Rawan Al-Mutairi^1^, Artak Tovmasyan^2^, Ines Batinic-Haberle^2^ and Ludmil Benov^1^***

**^1^ Department of Biochemistry, Faculty of Medicine, Kuwait University, Kuwait City, Kuwait**

**^2^ Department of Radiation Oncology, Duke University Medical Center, Durham, North Carolina, USA**

*** Correspondence:** Ludmil Benov: [lbenov@hsc.edu.kw](mailto:lbenov@hsc.edu.kw)

# Supplementary Figures and Tables

## Supplementary Figures

**Supplementary Figure 1** │ **Relationship between ZnTnHex-2-PyP concentration and *E. coli* inactivation**. Stationary phase *E. coli* culture was incubated for 30 min with PS and illuminated for 20 min at a fluence of 37 mW/cm^2^. Cell viability was determined by the MTT assay **(A)** and by plating and enumeration of colonies **(B)**. Results are presented as medians and 25/75 percentiles.

**Supplementary Figure 2** │**Effect of twenty cycles of sublethal photodynamic treatment and regrowth on susceptibility to aPDT.** After twenty cycles of treatment and regrowth, cultures were grown to stationary phase, cells were resuspended in PBS to OD_600_ = 0.5, incubated 30 minutes with 1 μM ZnTnHex-2-PyP in the dark and illuminated for 20 minutes. Cell metabolic activity was assessed by the MTT assay (**Panel A**) and ability to replicate was determined by plating and counting colonies (**Panel B**). All conditions were as in Figures 2 and 3. Cells that were not exposed to any treatment were considered 100% viable. Cell suspensions illuminated in the absence of ZnP (illuminated controls) and cultures incubated with 1.0 μM ZnTnHex-2-PyP but not illuminated (dark controls) were tested in parallel. Data is presented as medians and 25/75 percentiles.

**Supplementary Figure 3** │**Effect of growth phase on photodynamic inactivation.** Non-treated cells and cells exposed to ten cycles of sublethal photodynamic treatment and regrowth were grown to either mid-log (OD_600_ = 0.5 – 0.8) or to stationary phase. Samples were centrifuged, cell pellet was resuspended in PBS and photo-treatment with 1.0 μM ZnHex-2-PyP was performed. Metabolic activity was determined by the MTT assay.

**Supplementary Figure 4** │**Effect of ten cycles of mild photodynamic treatment and regrowth on photodynamic suppression of cell metabolism.** *E. coli* (GC4468) cell suspension was diluted in PBS to OD_600_ = 0.5, incubated for 30 minutes in the dark with 0.6 μM ZnTnHex-2-PyP and illuminated for 20 minutes at a fluence of 37 mW/cm^2^. Surviving cells were then regrown and subjected to the same procedure for ten consecutive times. Results show cell metabolic activity after the tenth cycle, determined by the MTT assay. Medians and 25/75 percentiles are presented.

**Supplementary Figure 5** │**Inactivation of antibiotic-sensitive and antibiotic-resistant *E. coli* and *S. aureus* strains by aPDT.** Cultures were grown to stationary phase, diluted in PBS to OD_600_ = 0.5, incubated for 30 min with 1.0 μM of ZnTnHex-2-PyP, and illuminated for 20 min at a fluence of 37 mW/cm^2^. Immediately after the illumination the MTT test was performed. Results are presented as percentage of MTT reduction compared to untreated controls.

## Supplementary Tables

**Supplementary Table 1** │**Antibiotic susceptibility of *E. coli* and *S. aureus* clinical isolates before and after ten cycles of sublethal photodynamic exposure**. After ten cycles of photodynamic treatment and regrowth, cultures of clinical isolates were tested to antibiotic susceptibility by the disk diffusion method. Original cultures not exposed to any treatment were analyzed the same way. The experiment was repeated three times. Zones of inhibition in millimeters are presented as mean ± S.D.

| **Bacterial strain** | **Chloramphenicol** | **Kanamycin** | **Tetracycline** | **Streptomycin** | **Gentamycin** | **Vancomycin** |
| --- | --- | --- | --- | --- | --- | --- |
| ***E. coli*** | 14.00 ±0.89 | 19.00±0.89 | 9.16±0.75 | 0.00±0.00 | 21.17±3.97 | 0.00±0.00 |
| ***E. coli 10 cycles*** | 15.16±0.75 | 18.83±0.75 | 8.67±0.51 | 0.00±0.00 | 19.17±0.75 | 0.00±0.00 |
| ***S. aureus*** | 29.50±1.87 | 0.00±0.00 | 20.83±1.83 | 16.17±1.16 | 9.83±0.75 | 17.50±1.04 |
| ***S. aureus* 10 cycles** | 28.50±1.87 | 0.00±0.00 | 20.33±1.03 | 15.66±1.75 | 9.16±1.47 | 16.83±0.75 |
